# Conduction system pacing on track to replace CRT? Review of current evidence and prospects of conduction system pacing

**DOI:** 10.3389/fcvm.2023.1220709

**Published:** 2023-08-15

**Authors:** Ahmed T. Moustafa, Anthony SL. Tang, Habib Rehman Khan

**Affiliations:** Schulich School of Medicine and Dentistry, University of Western Ontario, London, ON, Canada

**Keywords:** conduction system pacing, His bundle pacing, left bundle branch area pacing, LBBAP, biventricular pacing, CRT

## Abstract

Conduction system pacing (CSP) has been emerging over the last decade as a pacing option instead of conventional right ventricular (RV) pacing and biventricular (BiV) pacing. Numerous case reports, some observational studies and a few randomized control trials have looked at optimum pacing strategies for heart failure (HF) with left bundle branch block (LBBB) or cases where left ventricular (LV) dysfunction is anticipated due to chronic RV pacing (RVP). Evolution of pacing strategies from standard RVP to septal RVP, BiV pacing and now CSP have shown improving hemodynamic responses and possible ease of implantation of CSP systems. In this review article, we review the literature on the evolution of CSP and common scenarios where it might be beneficial.

## Atrioventricular conduction system—anatomy and physiological properties

**Figure 1:**
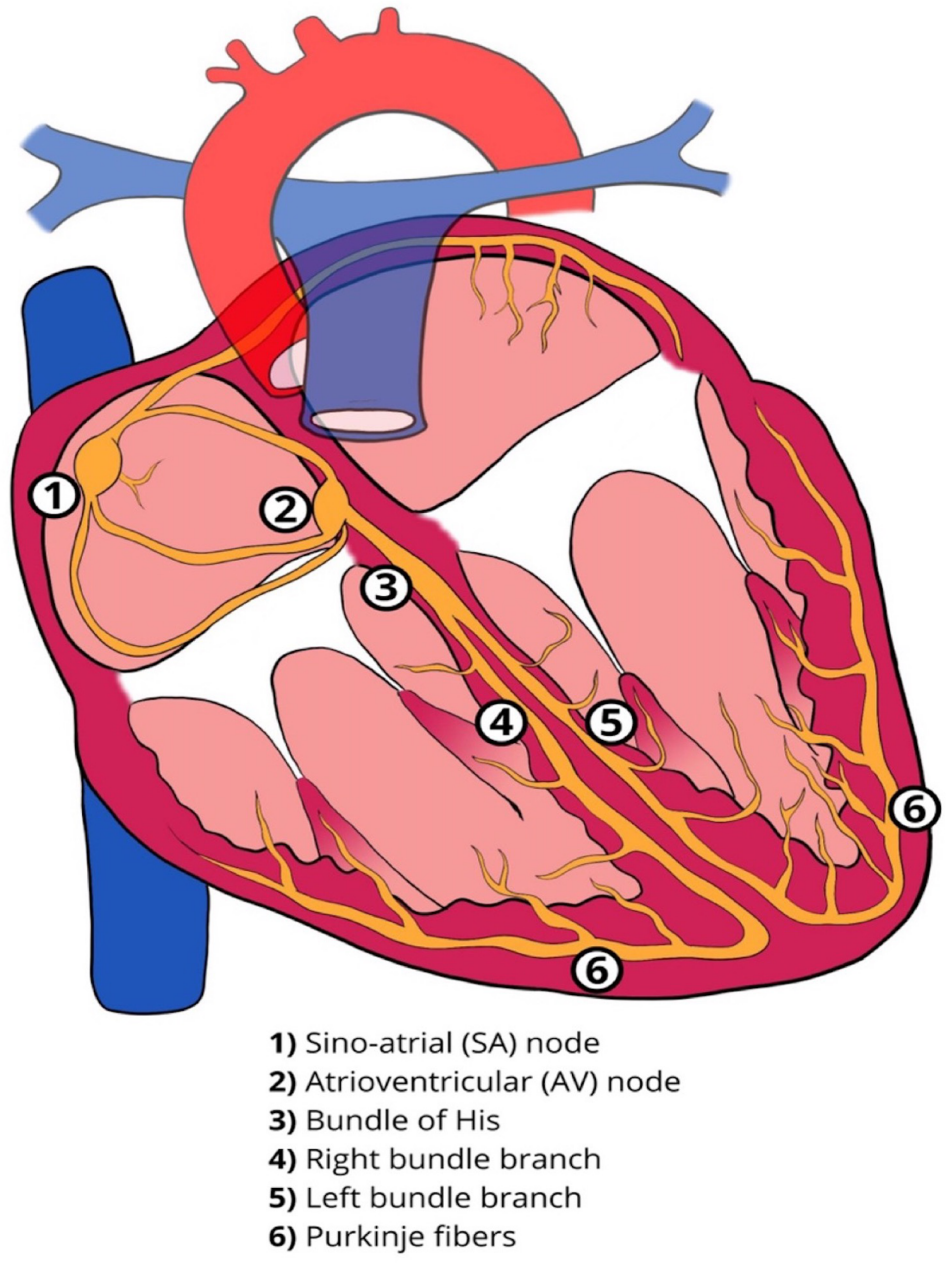
Normal cardiac conduction system.

**Figure 2:**
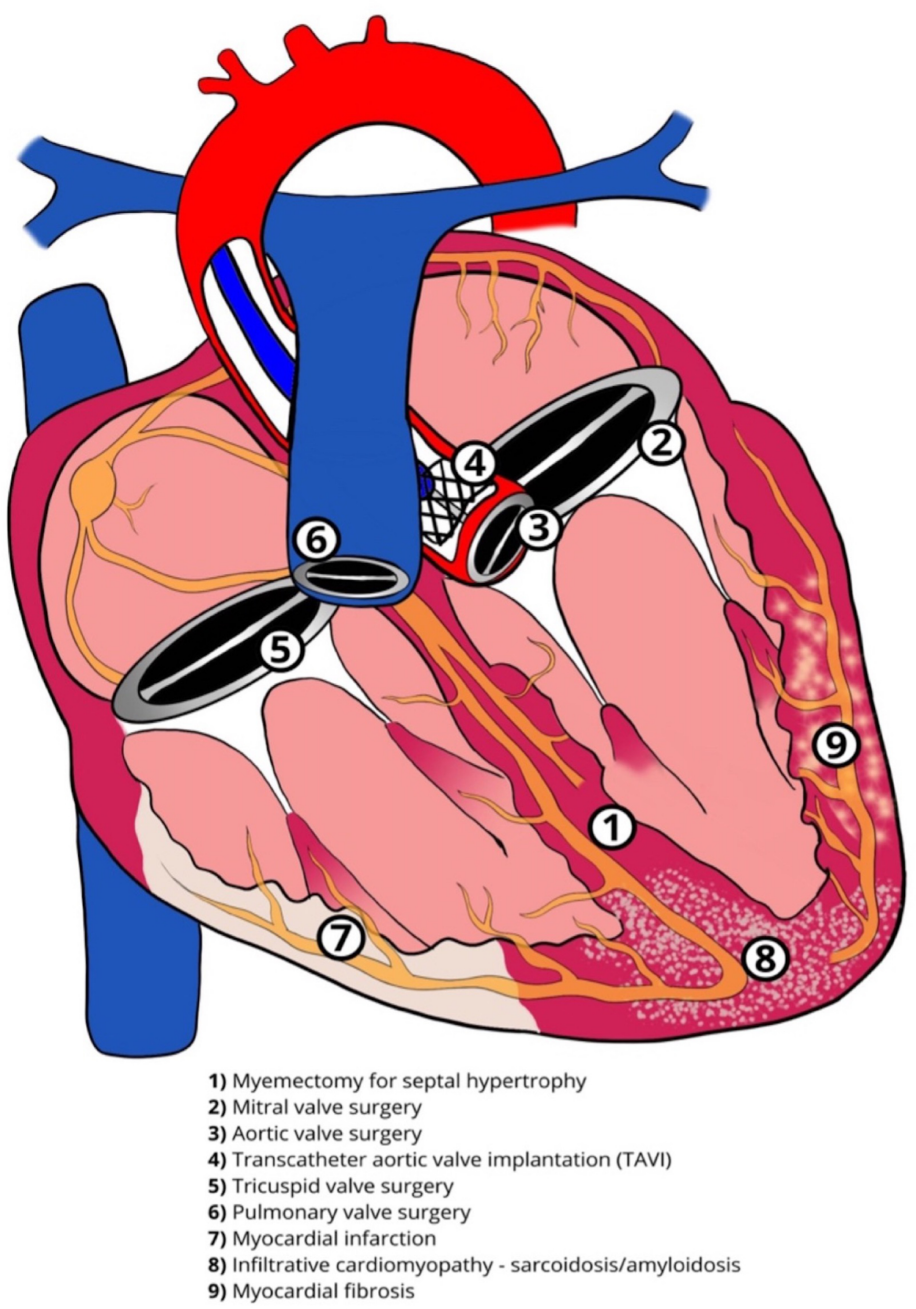
Cardiac conduction system abnormalities.

The cardiac conduction system comprises specialized cells with properties of automaticity and conduction. The sinoatrial (SA) node and the atrioventricular (AV) node can function as pacemakers with the ability of automaticity. The Bundle of His, approximately 18 mm long in an adult heart, traverses the right fibrous trigone commonly dividing into two specialized bundle branches (right and left bundle branch, RBB and LBB). These are encapsulated by a fibrous sheath that separates the specialized myocytes from the myocardium thus allowing rapid electrical conduction. At the distal branches of these bundles, there is absence of this fibrous sheath, allowing communication with the local ventricular myocardium resulting in myocardial contraction (Figure 1) (1). The LBB fibers are widely distributed and progressively broaden to create a subendocardial network, before dividing further into the fascicles of the LBB. This broad network further explains the feasibility of left bundle branch area pacing (LBBAP), unlike His bundles, where the target area for effective pacing is very narrow. Conduction abnormalities can occur in any segment of the conduction system from the SA node, AV node or the His-Purkinje system (Figure 2).

## Conduction tissue disease with bundle branch block

LBBB cause interventricular dyssynchrony with RV systole earlier than LV systole. It also contributes to intraventricular dyssynchrony, which is a result of myocyte-to-myocyte propagation of signal from RV to LV through the septum, with the earliest breakthrough of signals being the LV septum and the latest being the inferior-basal LV wall. This mechanical disparity results in a pre-systolic stretch of the late activated areas, which by way of Frank-Starling law results in an enhanced systolic, albeit late, contraction. As a result, systolic stress, strain, and myocardial oxygen consumption is increased in late activated regions and reduced in early activated regions, with subsequent loss of pumping efficiency (2–4).

Disruption of the conduction system can have varying detrimental effects. Early landmark data, such as the Framingham study, showed that new LBBB was associated with underlying coronary artery disease, cardiomyopathies, infiltrative diseases and heart failure (5). Incidental finding of LBBB in the presence of scar, as shown on cardiac magnetic resonance (CMR) imaging, has worse outcomes than those who have no scar (6). Bundle branch block (BBB) is deleterious on long-term cardiovascular outcomes and has higher mortality in those individuals with myocardial infarction or abrupt BBB following a percutaneous procedure such as percutaneous coronary intervention (PCI) or transcatheter aortic valve implantation (TAVI) (7). Meta-analysis of studies shows any form of BBB to have a higher association with mortality in patients presenting with acute heart failure (8). In patients with LBBB, there is an increasing evidence of LV diastolic impairment despite having a preserved LV systolic function resulting in elevated filling pressure and a rise in serum NT-proBNP levels (9).

Landmark clinical trials had shown that resynchronization with Biventricular Pacing—Cardiac Resynchronization therapy (BiV-CRT) improved quality of life, reduced LV remodeling, and reduced cardiovascular outcomes such as hospitalization and mortality in patients with impaired LV function in the setting of LBBB (10–12). Correction of RBBB with CRT in the setting of heart failure has not been shown to be as successful as shown in meta-analysis and is thought to be due to underlying comorbidities such as pulmonary hypertension (13).

There is minimal evidence supported by case reports to suggest that correcting the LBBB in preserved LV function has any long-term benefit (14). More studies are required to compare the effects of pacing different regions of the conduction system evaluating an improvement or maintaining LV function in these subsets of patients over long periods (15).

## Correction of conduction tissue disease

**Figure 3:**
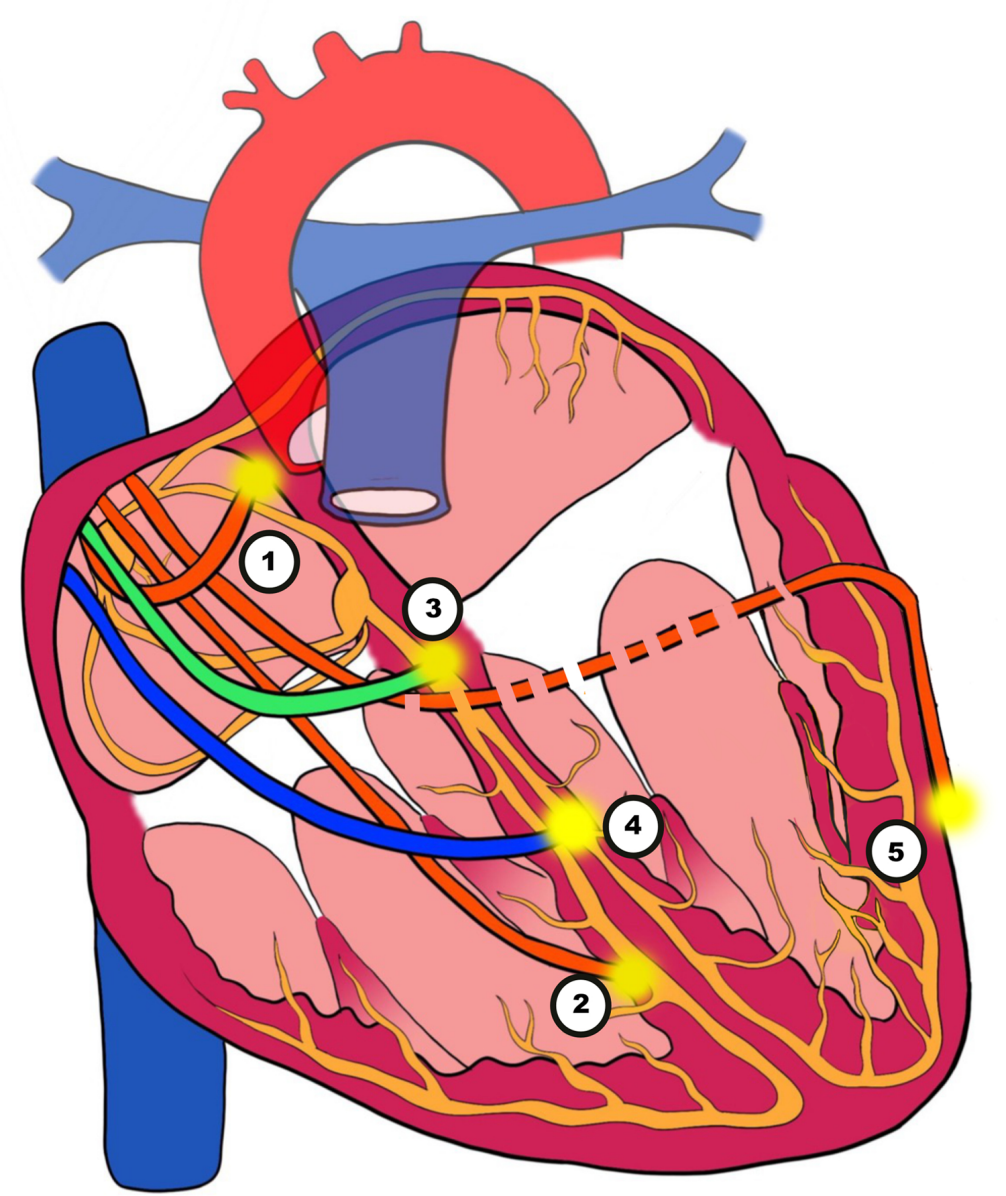
Illustration of pacing techniques. (1) Atrial pacing. (2) Right ventricular (RV) pacing. (3) His bundle pacing (HBP). (4) Left bundle branch area pacing (LBBAP). (5) Biventricular pacing (BVP) with an epimyocardial left ventricular lead via the coronary sinus (CS).

**Figure 4:**
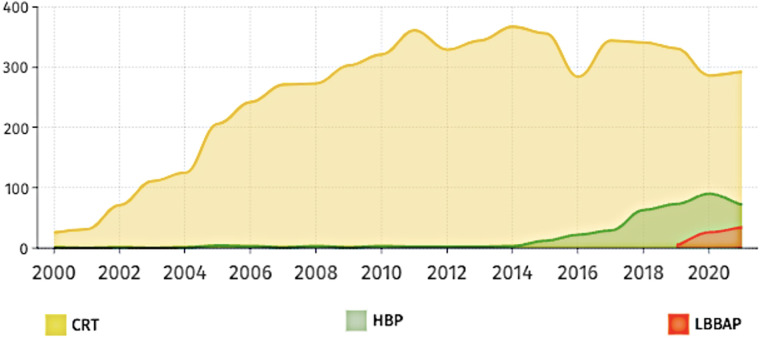
Number of publications over the last 20 years based on three main pacing techniques of HBP (HIS bundle pacing), LBBAP (left bundle branch area pacing) and CRT (cardiac resynchronization therapy).

Patients with symptomatic SA node disease and AV block will require pacemakers. The pacemakers can be RV-only, dual-chamber pacemakers, CRT, endocardial LV lead, HIS Bundle pacing (HBP) or LBBAP (Figure 3). Advancing technologies in this field have led to an increasing amount of literature being published particularly in the field of CRT, HBP and LBBAP (Figure 4).

## Pacing-induced cardiomyopathy and the need for resynchronization therapy

Right ventricular pacing (RVP) has saved the lives of a substantial number of people, especially those with sick sinus syndrome (SSS) and AV block such as complete heart block (CHB). However, chronic RVP has resulted in LV dysfunction in some patients.

The placement of the leads at the RV has deleterious effects on the normal functioning of the heart with the weakening of heart muscle, heart failure and risk of arrhythmia. Pacing-induced cardiomyopathy (PICM) from chronic high burden RVP has varied in definitions across studies, with several different thresholds for drop in LVEF identified. Most widely used definition is drop in LVEF to less than 40%–50%, with an absolute drop in LVEF of at least 5%–10%, with 10%–20% of patients with normal LVEF speculated to develop PICM from chronic high burden RVP (16–19). Permanent RVP results in interventricular dyssynchrony, due to a change in the normal activation of the LV. This effect is more prominent in patients with dilated LV and lower left ventricular ejection fraction (LVEF) (16, 18, 20). The altered pattern of ventricular activation, is responsible for intraventricular dyssynchrony, in a similar fashion as LBBB with earliest activation being the ventricular septum, and the latest being the inferior, basal wall of the LV. This contributes to impaired mechanical contraction and eventually PICM (21).

## Conduction system pacing with His bundle pacing and left bundle branch area pacing to normalize bundle branch block

Conduction system pacing (CSP) requires engaging the conduction system at either the level of the His bundle (HBP) or the left bundle branch (LBBAP) resulting in myocardial capture that is considered physiological. Physiological pacing through CSP has been evolving to avoid the unfavourable effects of RV pacing resulting in ventricular dyssynchrony (22–24).

**Figure 5:**
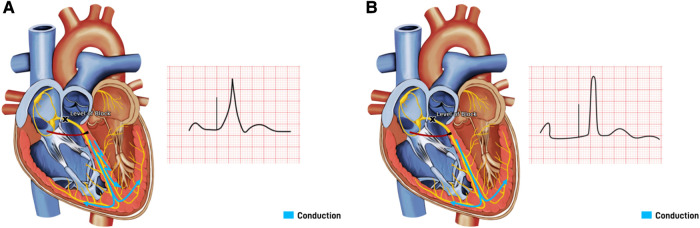
His bundle pacing with underlying block at the level of the atrioventricular (AV) node. (**A**) Non-Selective His bundle pacing with capture of both His bundle and the surrounding septal myocardium, resulting in a paced beat with a fusion pattern. (**B**) Selective His bundle pacing with capture of His bundle only, with a narrow complex paced beat.

HBP is considered the most physiological as it captures the proximal origin of the ventricular conduction system beyond the AV node, and proximal BBB can be corrected at its level due to the longitudinal dissociation theory (25, 26). This theory suggested that fibers from His bundle are predestined to fibers composing individual bundle branches. Therefore, HBP at levels of proximal blocks can allow for correction of BBB and result in cardiac resynchronization (25, 26). HBP can be selective (S-HBP) without simultaneous local myocardial capture or non-selective (NS-HBP) where a small portion of cardiac myocardium is simultaneously activated (Figure 5). The overall myocardial performance is comparable between S-HBP and NS-HBP, with early studies showing no clinical difference in outcomes from heart failure, hospitalization, or death, as well as activation pattern on echocardiography (27, 28). However, there is greater longevity of pacemakers with S-HBP due to higher excitability of HBP compared to myocardial excitability termed chronaxie, with His bundle exhibiting shorter chronaxie requiring a shorter pacing pulse width, further optimizing battery performance (29, 30). HBP preserves LVEF by ensuring electrical activation of distal Purkinje system and maintaining mechanical synchrony when compared to right ventricular septal pacing (RVSP) during mid-term follow-up in patients with AVB, narrow QRS, and LVEF >40% (31). HBP has also been shown to have a lower risk of inducing AF than conventional RVP (32). The 2018 AHA/ACC/HRS guidelines recommend physiological ventricular activation (CRT or HBP) in patients with AV block (class IIa indication), and HBP in patients with AV block at the level of the AV node (Class IIb indication), with mid-range LVEF (36%–50%) who require permanent pacing and expected to require pacing more than 40% of the time (33).

Despite HBP being the optimal pacing site for a physiological response, it has its limitations. The technique of HBP requires precision skills in targeting a small zone and is found to be challenging in patients with distorted anatomy due to dilated hearts, resulting in high fluoroscopic radiation exposure and a long duration of procedures. Oversensing the atrial and undersensing the ventricular signals is a frequent concern due to the anatomical location of HBP. The success rate of achieving HBP with predefined parameters varies between 76% and 96% depending on the operator's experience and the level of conduction system disease (34). There is also variability in the location of true His signals, necessitating different HBP lead tip trajectory (more atrial vs. ventricular), as shown in the IMAGE-HBP study, by identifying the HB anatomic landmarks on the basis of CT imaging and lead tip location (35). Despite the successful deployment of HBP, the pacing threshold is higher than conventional RVP and can result in premature battery depletion, loss of capture, early lead revisions, and associated risks from repeated generator replacements. Mean number of years for HBP generators to reach End Of Life (EOL) necessitating a generator replacement is 5.9 ± 2.1 (36). When compared to standard RVP, the need for lead revisions (6.7% vs. 3%) and for generator change (9% vs. 1%) were higher in the HBP group at 5 years (37). Out of 844 patients with HBP, 199 (23.5%) patient's thresholds increased more than 1.0 V in respect to implant value, leading to HBP interruption in 51 patients, and necessitating reintervention (lead revision or replacement) in 31 patients (36). Moreover, in cases of LBBB when the level of conduction block is more distal, HBP cannot correct the LBBB.

**Figure 6:**
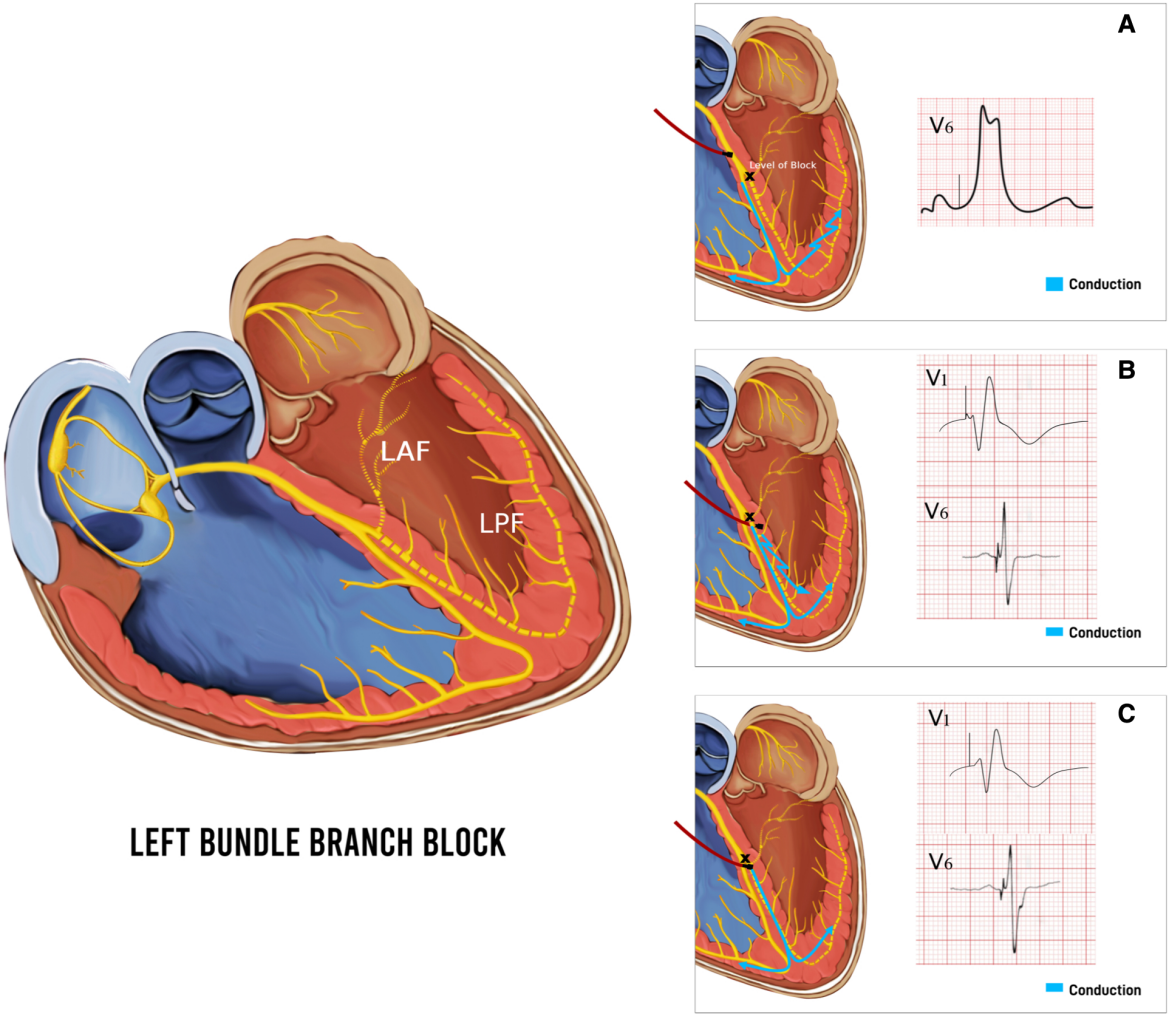
Baseline left bundle branch block (LBBB) and respective QRS morphology and duration with pacing different parts of the conduction system. (**A**) Pacing above the level of block at the His Bundle with the paced beat resembling LBBB. (**B**) Non-Selective left bundle branch pacing (LBBP) with capture of both left bundle branch (LBB) and surrounding myocardium resulting in a paced beat with fusion pattern and atypical RBBB pattern with qR in V1. (**C**) Selective LBBP with capture of LBB only, resulting in a typical wider RBBB pattern with rSR′ in V1. LAF, left anterior fascicle; LPF, left posterior fascicle.

LBBAP technique was first described by Huang et al. in 2017 by capturing the LBB area deep into the RV septum in a patient with heart failure and LBBB (2). LBBAP has emerged as a suitable alternative to HBP due to lower pacing thresholds, higher sensing amplitudes, and more stable lead positions alongside providing physiological pacing (38, 39). Depending on the area captured with pacing, LBBAP could either be LBB pacing (LBBP) with both selective and non-selective LBB pacing, as well as LV myocardial-only septal pacing (LVSP), each defined with their unique diagnostic criteria (38, 39). LBBP can be achieved by engaging the conduction system of the LBB along its left-sided septal course by deploying the lead deep into the RV septum to reach the LBB (2). LBBP results in more selective pacing of LBB and restores a synchronized LV electrical activation more readily due to a broader area to implant the pacing lead. At a higher pacing output, LBBP usually results in non-selective LBBP, capturing both the LBB and the surrounding myocardium. Whereas at a lower pacing output, only the LBB is captured if the pacing lead tip is appropriately positioned to capture the LBB, resulting in selective LBBP (Figure 6). LVSP share the same approach, implanting the lead into the left ventricular septum via a transseptal approach, albeit shallower for LVSP (42). LVSP entails capture of LV septal myocardium in contrast to the direct LBB conduction system capture with LBBP, is a common procedural outcome during LBBP due to the inability to accurately distinguish them sometimes, or due to the implanters experience (43). Despite the subtle differences in LV activation, QRS morphology and duration, long term outcomes of LVSP vs. LBBP are unknown and may differ. A subgroup analysis of LOT-CRT study showed better echocardiographic, electrocardiographic, and clinical outcomes in LBBP compared to LVSP (44). LBBP continues to be a more favorable outcome over LVSP in heart failure patients to achieve pacing closest to physiological activation.

LBBAP has shown to be safe and effective in LBBB patients with LV impairment to maintain or improve cardiac function compared to RV pacing and BiV-CRT pacing, while taking a shorter time and less radiation exposure to the patient than HBP (45). Early small retrospective and prospective studies showed promising results with the evolution of LBBAP to correct LBBB and improve heart failure through a reduction in QRS duration (QRSd) resulting in quicker LV activation time, with a low, stable pacing capture threshold (46, 47).

LBBAP represents a natural evolution of conduction system pacing to overcome the challenges posed by the current limitations of HBP (48). There are some safety concerns however associated with LBB pacing which include LV perforation, acute lead dislodgement, and RBB injury necessitating a temporary pacing lead for backup in some patients with LBBB (49–52). Coronary artery injury, specifically septal branches of the left anterior descending artery (LAD) is a risk of LBBAP (53). One other limitation of LBBAP is the inability of the lead to penetrate deep into the septum due to underlying fibrosis in some patients with ischemic or non-ischemic cardiomyopathies (54). The long-term safety profile, lead performance, and risks associated with the extraction of the deep septal lead needs to be determined (55). The efficacy of LBBAP for cardiac resynchronization requires investigation in prospective randomized clinical trials.

For instances where adequate narrowing of the QRS cannot be achieved by HBP due to distal conduction tissue disease, His optimized CRT (HOT-CRT) was considered an alternative for narrowing QRS by fusion of HBP and LV pacing via the coronary sinus (56). Similarly, LBBAP-optimized CRT (LOT-CRT) allows for fusion between LBBAP and LV pacing through the coronary sinus (57). Both HOT-CRT and LOT-CRT techniques have shown significant improvements in QRSd, LVEF, and reduction in NT-proBNP and HF symptoms (44, 58).

Current recommendations for CSP (HBP or LBBAP) from the 2023 HRS/APHR/LAHRS guidelines include patients with indications for pacemaker therapy with anticipated ventricular pacing ≥40% and an LVEF of 36%–50% (class IIa) or LVEF >50% (class IIb). CSP maybe considered if less than 40% pacing is anticipated, with LVEF of 36%–50%, with or without a LBBB (class IIb), whereas only LBBAP maybe considered if LVEF is >50% (class IIb). CSP maybe also considered in HF patients with LBBB, LVEF 36%–50%, QRSd ≥150 ms and NYHA class II-IV (class IIb), or if effective CRT cannot be achieved with BiV pacing and LVEF ≤35% (class IIa). In patients with non-LBBB, LVEF ≤35%, QRSd 120–149 and NYHA class III–IV, CSP could be considered (class IIb) (59).

## Biventricular pacing resynchronization of ventricles and limitations

BiV pacing (BVP) was developed in the early 2000s by pacing both ventricles resulting in fusion and correcting BBB in the management of heart failure (60). Conventional BiV-CRT is achieved by placing a lead in an epicardial coronary vein. Evidence for BiV-CRT stems from the MIRCLE trial, the first trial showing improved exercise tolerance, heart failure symptoms and quality of life (61). Subsequent trials (CARE-HF, COMPANION, REVERSE, MADIT-CRT and RAFT) showed similar promising outcomes (10, 62–65). Currently, BVP is an established guideline treatment termed cardiac resynchronization therapy (CRT) for patients with heart failure (LVEF <35%), LBBB and on optimal recommended heart failure drugs (66–68).

BiV-CRT has been shown to reverse LV dysfunction by narrowing QRS, and improving hemodynamic response, even in patients with chronic RVP and mild heart failure (69–71). Several pathways have been identified by which BiV-CRT improves cardiac function and heart failure symptoms. Most importantly is correction of LBBB, improving both inter- and intraventricular dyssynchrony, eliminating the disparities in the timing of shortening of earlier activated regions with reciprocal stretch of late activated regions (72). Derivate of pressure over time maximum (dP/dT_max_) of the LV, is one of the oldest measures of LV global contractility and is a good index of ventricular performance. Shortening of an inappropriately long AV delay by CRT results in an earlier pressure development in the LV due to pre-excited pacing, increasing the pulse pressure and LV dP/dT_max_ (73–75).

BiV-CRT has its challenges and complications. Besides the problems associated with conventional pacing, there are several others, like the problems associated with the LV lead insertion due to coronary sinus anatomy and perforation, phrenic nerve stimulation, and displacement of leads, along with longer procedure times with subsequently increased risk of infections (76, 77).

Despite the success, 30%–45% are considered non-responders to CRT and do not benefit, albeit there is a lack of a universally accepted definition for non-responders (78). Patients with a history of Atrial fibrillation (AF) may not have good outcomes with the BiV-CRT alone and will require >98% BVP achieved by medications or by catheter ablation of the AV node (79, 80). Another predictor identified that is associated with poor response to CRT is a QRS morphology with typical LBBB being more responsive to CRT compared to atypical LBBB or RBBB (70). The location of the epicardial LV lead also plays an important role explained by pacing not being at the most delayed region or presence of scar at the LV pacing site (81, 82). With apical epicardial placement resulting in worse clinical outcomes compared to placement at the basal to mid-myocardial segments of the lateral wall in addition to variable activation wavefront vectors and velocities resulting in altered fusion (83). The advantages of multisite pacing in the LV has also not improved response rates and are likely due to large areas of fibrosis and not the anatomical site of pacing (84, 85).

A “CRT team” using CMR and longitudinal myocardial strain to identify a target area for optimal epicardial LV lead placement prior to implantation, defined as the most delayed and still viable region, showed a high response rate with only 7% non-responders and no negative responders (86). In areas of progressive scar, phrenic nerve capture, and higher rates of coronary sinus lead dislodgements, quadripolar leads have shown advantageous to bipolar leads in preserving CRT response and avoiding premature battery depletion (87–89).

LV endocardial pacing was designed to offer another alternative option for LV resynchronization with a greater choice of the site of LV pacing without the restriction of implanting in coronary venous tributaries. Approaches for LV endocardial pacing include atrial transseptal route and across the mitral valve, via the interventricular septum, via a transapical route, and lastly wireless LV endocardial pacing. The main limitation to LV endocardial pacing is placement of the lead in the systemic circulation with increased risk of thromboembolism and the requirement for long-term anticoagulation (90). In addition, the LV endocardial pacing approach via the intra-atrial septum passing through the mitral valve has its challenges, such as impeding mitral valve closure and increasing the risk of degeneration and infective endocarditis (91). The transapical implantation method of endocardial LV pacing is beneficial as it avoids the mitral valve and transseptal route. However, it has only been described in case reports and small series (92, 93). Due to its limitations, LV endocardial pacing has not been sought after as an alternative to conventional BVP.

## His bundle pacing compared with biventricular pacing

CSP in the form of HBP is used in patients with heart failure with reduced LVEF (HFrEF) and broad QRS and has shown to have promising results as supported by a recent meta-analysis (94). There have been few case reports, observational and randomized control trials comparing HBP to CRT, showing a reduction in QRSd, LV volumes and improvement in LVEF (94, 95). HBP, compared to BiV pacing, was superior in symptomatic AF patients undergoing AV node ablation, with moderately reduced LVEF (≥35% and <50%) and a narrow QRS (≤120 ms), with a statistically significant reduction in indexed LV volumes and an increase in LVEF (96). Similarly, the ALTERNATIVE-AF trial demonstrated modest improvement in LVEF with HBP compared to BiV pacing in patients with symptomatic persistent AF, reduced LVEF (≤40) and a narrow QRS (≤120 ms) (97). However, further adequately powered trials are necessary to determine whether these improvements in LV function can translate to improvements in clinical endpoints.

## Left bundle branch area pacing compared with biventricular pacing

CSP in the form of LBBAP has evolved after the era of HBP mostly due to the latter's limitation with successful His engagement, increasing capture threshold during follow-up, proximity to atrium resulting in oversensing and low sensing amplitude at the His bundle location. In contrast, LBBAP regions typically have higher sensing, lower capture thresholds and similar paced QRS durations. Compared to BiV pacing, LBBAP showed improved symptoms, LVEF and reduction in QRS and LV volumes (55, 94, 98, 99). The randomized trial LBBP-RESYNC, demonstrated a greater improvement of LVEF, reduction in indexed LV systolic volume with LBBAP compared to BiV pacing in patient with symptomatic HF, LBBB and an LVEF ≤40, with comparable improvement in functional status with both pacing modalities (100). A metanalysis of only four available non-randomized controlled trials of LBBAP vs. BiV-CRT showed a significantly shortened QRSd (MD: −29.18 ms, 95% CI: −33.55 to 24.8, *P* < 0.001), improved LVEF (MD: 6.93%, 95% CI: 4.69 to 9.17, *P* < 0.001), reduced LV end-diastolic dimension (MD: −2.96 mm, 95% CI: −5.48 to −0.44, *P* = 0.02), improved NYHA class (MD: −0.54, 95% CI: −0.84 to −0.24, *P* < 0.001), and higher echocardiographic and clinical response rate (48). Two non-randomized trials comparing LBBAP and BiV pacing, one with 371 patients, and an international multicentre trial with 1,778 patients, showed a significant reduction in the primary outcome, which was a composite outcome of both HF hospitalization and all-cause mortality, driven by a greater reduction in HF hospitalizations without a significant difference in all-cause mortality or long-term complications in the LBBAP group, with greater LVEF improvement, improved functional status, and a significant reduction in procedural and fluoroscopy times (101, 102). In a non-randomized trial, with 12 months follow-up, patients with LBBB, LVEF ≤35% and heart failure had better electromechanical resynchronization with LBBP compared to optimized BiV pacing with adaptive algorithm (BVP-aCRT), with a significant reduction in QRSd (126.54 ± 11.67 vs. 102.61 ± 9.66 ms, *P* < 0.001). Furthermore, LBBP demonstrated higher clinical and echocardiographic response, especially higher super-response (≥20% absolute increase or LVEF ≥50%) compared to BVP-aCRT (103). LBBAP is a promising alternative over BiV-CRT, however, high quality randomized controlled trials with longer terms are essential for validation.

## Future considerations

The rapid acceptance and evolution of CSP along with its safety has led to rapid growth of research in multiple facets being explored. Current randomized recruiting studies on CSP listed on clinicaltrials.gov include CONSYST-CRT (NCT05187611) compares CSP to BiV-CRT in 130 patients with indications for CRT, and Left vs. Left (NCT05650658) comparing both pacing strategies in 2,136 patients with LVEF <50%, RAFT-Preserved (NCT04582578) compares CSP to BiV-CRT in HF with preserved ejection fraction, RAFT P&A (NCT05428787) compares LBBAP to BiV-CRT in patients with AF underdoing a pace and ablate strategy, CSP-SYNC (NCT05155865), HOT-CRT (NCT04561778), PHYSPAVB (NCT05214365) compares CSP versus conventional RV pacing, LEAP (NCT04595487) compares CSP to RV pacing, LEAP-Block (NCT04730921) compares LBBAP to RV pacing in patients with AV block, HIS-PrEF (NCT04529577) compares RV pacing to HBP in HFrEF, HIS-alt_2 (NCT04409119) compares CSP to BiV-CRT in HFrEF with LBBB, LBBAP-AFHF (NCT05549544) compares LBBAP with BiV pacing in patients requiring an AV node ablation in LVEF <50%, LEFT-BUNDLE-CRT (NCT05434962) compares LBBAP to BiV-CRT in HFrEF. Some of these studies are reviewing the clinical effectiveness of CSP to current conventional and gold standards such as BiV-CRT with outcomes targeting electrical (QRS), function (LVEF) and biochemical changes (NT-proBNP). Other studies are looking at mechanisms of clinical response, and myocardial activation sequence using CSP compared to BiV.

## Conclusion

Conduction tissue pacing in HIS Bundle pacing and LBBAP is feasible, safe, and quicker, with results comparable to cardiac resynchronization therapy. Extensive studies are required to directly compare the long-term clinical effectiveness of conduction system pacing against CRT.
